# Single-cell analysis reveals dysregulated inflammatory response in peripheral blood immunity in patients with acute respiratory distress syndrome

**DOI:** 10.3389/fcell.2023.1199122

**Published:** 2023-05-22

**Authors:** Jingjia Mo, Yanli Yang, Jihua Feng, Yanhua Lei, Suhong Huang, Weiluan Cen, Shanshan Wei, Hao Huang, Junyu Lu, Jianfeng Zhang

**Affiliations:** ^1^ Department of General Practice, The Second Affiliated Hospital of Guangxi Medical University, Nanning, China; ^2^ Department of Emergency Medicine, The Second Affiliated Hospital of Guangxi Medical University, Nanning, China; ^3^ Intensive Care Unit, The Second Affiliated Hospital of Guangxi Medical University, Nanning, China

**Keywords:** acute respiratory distress syndrome, single-cell RNA sequencing, sepsis-induced ARDS, pneumonia-induced ARDS, immune dysregulation, oxidative stress, apoptosis

## Abstract

**Introduction:** Acute respiratory distress syndrome (ARDS) remains a major clinical challenge for patients in intensive care units. Determining the differential mechanisms underlying ARDS with different etiologies is a key goal to improve the effectiveness of ARDS therapy. Despite growing evidence that different immune cell types are involved in ARDS, the role of altered immune cell subpopulations in disease progression is unelucidated.

**Methods:** In this study, we combined scRNA-seq and bulk-level sequencing to analyze the transcriptomes of peripheral blood mononuclear cells from healthy volunteers and patients with septic ARDS (sep-ARDS) and pneumonic ARDS (PNE-ARDS).

**Results:** Our data revealed differential alterations at the cellular and molecular levels and within biological signaling pathways in ARDS with different etiologies. The dynamics of neutrophils, macrophages (Macs), classical dendritic cells (cDCs), myeloid-derived suppressive cells (MDSCs), and CD8^+^ T cells varied significantly among groups of different samples, with neutrophils and cDCs at higher, and Macs at significantly lower, amounts in the patients with sep-ARDS. Furthermore, MDSCs were highly enriched only in the sep-ARDS patients, whereas a higher abundance of CD8^+^ T cells was observed in patients with PNE-ARDS. In addition, these cell subpopulations were found to be significantly involved in apoptosis, inflammatory, and immune-related pathways. In particular, a significant enhancement of the oxidative stress response was observed in the neutrophil subpopulation.

**Conclusion:** Our study shows that the composition of cells involved in the main peripheral circulation differs in patients with ARDS with different etiologies. Studying the role and mechanism of action of these cells during ARDS will provide new opportunities for the treatment of this condition.

## 1 Introduction

Acute respiratory distress syndrome (ARDS) is a clinical syndrome defined as a type of respiratory failure caused by various injuries such as pneumonia, sepsis, trauma, and certain viral infections and results in significant morbidity and mortality (Bellani et al., 2016; Meyer et al., 2021). ARDS is usually accompanied by overactivation of the immune system, resulting in inflammation of the lungs, pulmonary edema, alveolar damage, and often respiratory failure (Stapleton et al., 2005; Fan et al., 2018). Mortality due to ARDS has remained high since the disease was discovered, and it varies between different regions and hospitals. The LUNG SAFE study presented a hospital mortality of 40% in 50 countries (34.9%, 40.3%, and 46.1% for mild, moderate, and severe illness, respectively) (Bellani et al., 2016). Despite intensive research and several clinical trials performed in recent years (Acute Respiratory Distress Syndrome et al., 2000; Guerin et al., 2013), treatment options remain limited. So far, there is no specific pharmacotherapy for the treatment of ARDS. The most effective management remains ventilatory supportive therapy, and a consensus regarding the optimal strategy still needs to be reached (Meyer et al., 2021).

Despite the presence of other triggers that precipitate systemic inflammatory responses and ARDS development, such as pneumonia, aspiration, trauma, pancreatitis, or multiple blood transfusions, sepsis is the leading cause of ARDS, accounting for 32% of ARDS etiology (Bersten et al., 2002). Based on the clinical data, sepsis-associated ARDS has a lower incidence in patients (approximately 6%–7% in Western countries) than sepsis or ARDS alone, but patients with sepsis-related ARDS exhibit worse clinical outcomes (Mikkelsen et al., 2013; Kim and Hong, 2016). Additionally, bacterial pneumonia and viral pneumonia frequently cause ARDS, with pandemic and surge peaks in the global incidence of ARDS associated with the influenza and novel coronavirus outbreaks that have been prevalent in recent years (Wang et al., 2020; Wu and McGoogan, 2020). However, researchers have yet to elucidate the multifactorial and differential mechanisms underlying ARDS with different etiologies; determining these mechanisms remains a key goal to improve the effectiveness of ARDS therapy.

Previous studies have compared patients with sepsis- and acute lung injury-induced ARDS to identify biomarkers with diagnostic and prognostic significance for ARDS of different etiologies (Tang et al., 2007; Feng et al., 2021). We previously identified dysregulated genes in the evolutionary process from healthy to sepsis to sepsis-induced ARDS by analyzing datasets from the Gene Expression Omnibus (GEO) database (Zhang et al., 2019). These studies deepened our understanding of the mechanisms underlying ARDS with different etiologies. However, elucidating the ARDS mechanisms at the transcriptome level remains to be achieved (Zhao et al., 2021); single-cell RNA sequencing (scRNA-seq) can overcome this limitation by unbiased sequencing of transcripts from individual cells and has the potential to provide deeper insights into alterations at the cellular level in ARDS. Despite growing evidence that different types of immune cells are involved in the course of ARDS, including regulatory T cells (Tregs; anti-inflammatory and regenerative effects), macrophages (Macs; cell polarization), neutrophils (reticular deposition), and T helper (h)-17 cells (pro-inflammatory response) (Matthay et al., 2019; Wong et al., 2019), the specific mechanisms of their action remain obscure.

To better understand the dysregulated immune response associated with ARDS, we analyzed the transcriptomes of peripheral blood mononuclear cells (PBMCs) collected from patients with sepsis- and pneumonia-induced ARDS using scRNA-seq combined with transcriptome sequencing technology (RNA-seq) and whole genome bisulfite sequencing (WGBS) and compared them with that of a healthy control. Our results show how the study of the role and mechanisms of action of specific immune cells that we found to be involved in ARDS development and progression can lead to new opportunities for ARDS treatment.

## 2 Materials and methods

### 2.1 Human sample collection

Peripheral blood samples were collected from one patient with pneumonic ARDS (PNE-ARDS), two patients with sepsis-induced ARDS (sep-ARDS), and one healthy volunteer at the Second Affiliated Hospital of Guangxi Medical University. All patients met the Berlin definition of ARDS, did not receive organ transplantation, did not have active malignancies, and were not treated with systemic immunosuppressants or systemic glucocorticoids. See Supplementary Table S1 for clinical information of the sample. The study was approved by the Research Ethics Committee of The Second Affiliated Hospital of Guangxi Medical University (Nanning, China) in accordance with the declaration of Helsinki, and all participants provided written informed consent before study commencement.

Mononuclear cells were isolated using a lymphocyte isolation solution (Ficoll Isolation Solution: anticoagulant = 1:1). The cell concentration was adjusted to 700–1,000 cells/μL by dilution in phosphate-buffered saline (PBS) containing 0.5% bovine serum albumin (BSA), and the samples were cryopreserved in 90% fetal bovine serum/10% dimethylsulfoxide (DMSO) and stored in liquid nitrogen.

#### 2.2 cDNA library preparation and scRNA-seq

Sample preparation and cDNA library construction were performed as described in the 10× Genomics Single Cell 3′v3.1 Kit User Guide. The cDNA product and library concentrations were detected according to the Qubit 4.0 fluorescence quantitative instrument, and the insertion fragment size of the cDNA library was detected using Qseq400 biological analyzer. Finally, the sample library was sequenced using a NovaSeq 6000 instrument of the Illumina platform. After recognizing the Casava base, the obtained original image file was converted into a sequence file and stored in fastq format. Then, 10× genomics official software CellRanger was used to compare and quantify the sequencing data. Single-cell transcriptome sequencing was performed by Biomarker Technologies Corporation (Beijing, China). We evaluated the violin plot distribution of the number of unique molecular identifiers (nUMI) as well as the total number of genes detected per cell (nFeatures) in all samples. Cells with gene expression numbers lower than 200 were filtered and cells with more than 10% mitochondrial transcript content were removed. These quality control metrics aimed to filter out cells with poor viability and quality.

### 2.3 DNA library construction and whole genome bisulfite sequencing

After sample testing, a negative control (lambda DNA) of 0.5 ng was added, and the genomic DNA was first randomly sheared via sonication to 200–300 bp using Covaris S220, followed by treatment with bisulfite using Accel-NGS^®^ Methyl-Seq DNA Library Kit (Swift Biosciences). Following treatment, unmethylated C is converted to U (T after PCR amplification), whereas methylated C remains unchanged. After library construction, initial quantification was performed using Qubit 2.0 (Life Technologies, Carlsbad, California, United States) to dilute the library to 1 ng/μL, followed by determination of the insert length of the library using Agilent 2100 (Agilent Technologies, Palo Alto, CA, United States). The effective concentration of the library was accurately quantified using quantitative real-time polymerase chain reaction (qRT-PCR) (effective library concentration >2 nM) to ensure the quality of the library. Subsequently, the libraries were pooled according to the effective concentration and the target downstream data volume and subsequently subjected to high-throughput sequencing (Illumina HiSeq/NovaSeq).

### 2.4 Transcriptome sequencing (RNA-seq)

Total RNA was extracted from each cell group using Trizol reagent (Thermo Fisher Scientific, Waltham, MA, United States) according to the manufacturer’s instructions. RNA sample quality was assessed using Nanodrop 2000 (ThermoFisher) and BioAnalyzer 2100 (Aglient Technologies). The RNA-seq libraries were sequenced using a TruSeq Stranded mRNA library preparation kit [(Illumina, San Diego, CA, United States)] and constructed as well as sequenced using HiSeq × Ten (Illumina) by BioMarker (Beijing, China). The RNA-seq reads were trimmed, filtered, and quality-controlled using FastQC (Babraham Institute) tools. Reads per kilobase per million mapped reads (RPKM) were then calculated using Hisat2 (Kim et al., 2015).

#### 2.5 Differential gene expression analysis

At the single-cell level, differentially expressed genes (DEGs) among PBMCs from the healthy volunteer and sep-ARDS and PNE-ARDS patients were identified using the “FindAllMarkers” function, and differences with a P-adjust <0.05 were considered significant. The normalizeBetweenArrays function in the limma package was used to normalize the gene expression profile. In addition, differential gene expression analysis was performed using the limma package in R language (Ritchie et al., 2015), and DEGs and differentially methylated genes (DMGs) at the bulk level were obtained for the healthy control, septic ARDS patients, and pneumonic ARDS patients, while *p* < 0.05 and |log fold change (logFC)| > 0.5 were considered significant.

### 2.6 Construction of single-cell atlas

Single-cell data were merged using the IntegrateData function (Butler et al., 2018) of the Seurat package (Stuart et al., 2019) in R to perform cell clustering analysis with default parameters. The clustering results were downscaled and visualized (Becht et al., 2018) based on the uniform manifold approximation and projection (UMAP) technique and subsequently projected onto a two-dimensional image defined as a single-cell atlas. In addition, the FindAllMarkers function of the Seurat package was used to identify genes specifically expressed in each cell cluster, with *p* < 0.05 and |logFC| > 0.5 being considered to indicate significance. In addition, cell types were annotated according to known markers and were re-clustered in this study for cell types associated with immune dysregulation in the ARDS microenvironment.

### 2.7 Functional and gene enrichment analyses

To explore the biological processes (BPs) and pathways in which the marker for each cell cluster is involved, the R package clusterProfiler (Yu et al., 2012) was used to perform Gene Ontology (GO) and Kyoto Encyclopedia of Genes and Genomes (KEGG) enrichment analyses, with *p* < 0.05 being considered to indicate significance.

### 2.8 Single-cell pseudo-time analysis

Based on changes in gene expression of different cell subpopulations over time, we used the Monocle 3 package in R (Trapnell et al., 2014) to reconstruct the differentiation developmental trajectory of dysregulated immune cell types from the peripheral circulation in ARDS patients to explore changes in dysregulated immune cells during ARDS development.

### 2.9 Gene regulatory network (GRN) analysis

Using the Python module tool pySCENIC (Van de Sande et al., 2020), we comprehensively reconstructed the transcription factor-centered GRN to further explore the regulatory mechanisms of dysregulated immune cell types. Binding motifs for transcription factors were obtained from the JASPAR database (https://jaspar.genereg.net/).

### 2.10 Data analysis and statistics

All statistical analyses were performed in the Bioinforcloud platform (http://www.bioinforcloud.org.cn), which was applied by calling the appropriate R package. Comparisons between the two groups were performed using Student's t test and correlation coefficients were calculated using Spearman analysis. *p* < 0.05 was considered significant.

## 3 Results

### 3.1 Global single-cell atlas of ARDS

**FIGURE 1:**
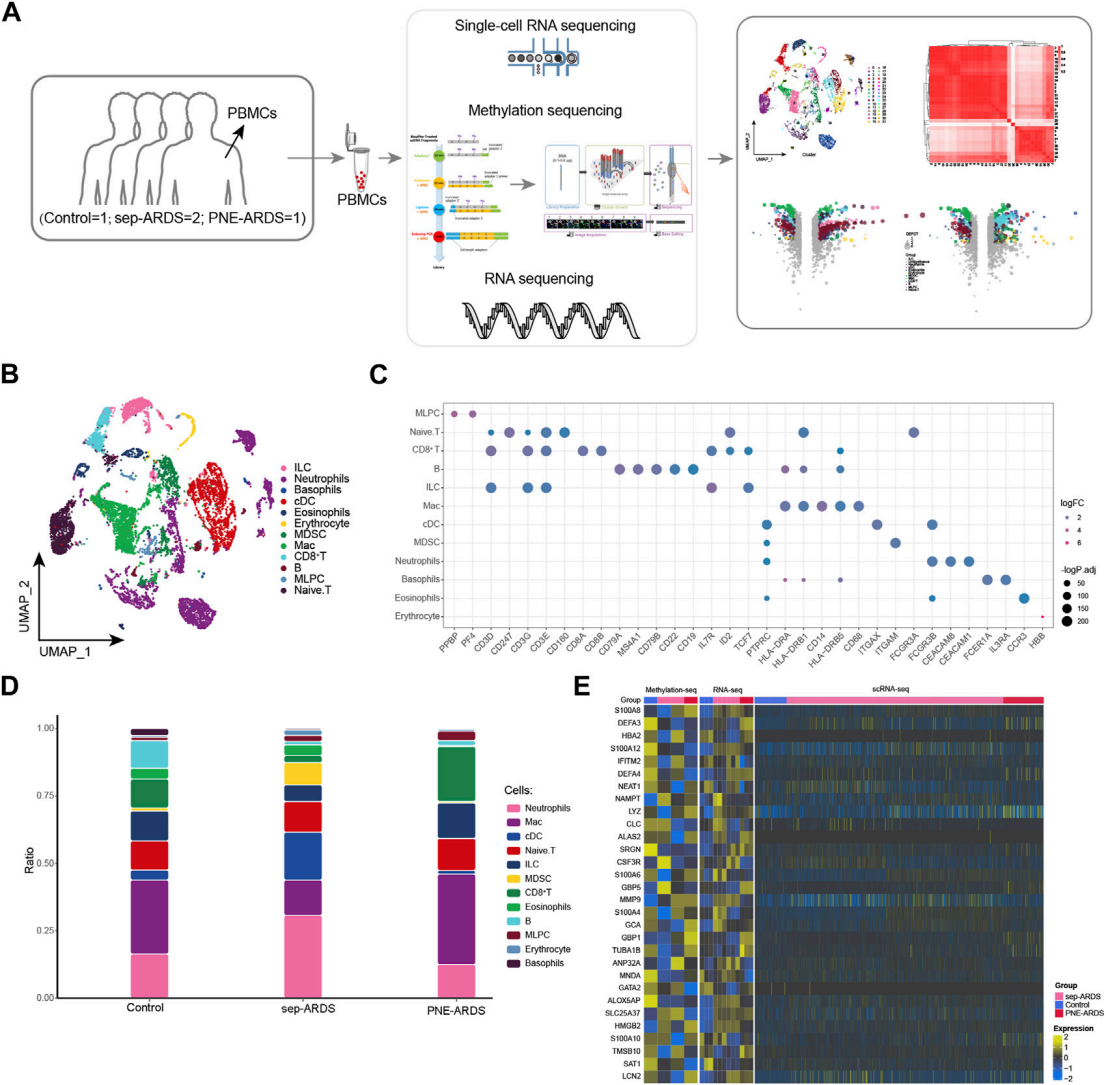
Global single-cell atlas of acute respiratory distress syndrome (ARDS). **(A)** The workflow of this study. **(B)** Single-cell atlas showing the global single-cell landscape of ARDS. **(C)** Bubble map showing cell-specific markers guiding cell annotation. **(D)** Bar graphs showing differences in cell type abundance among the healthy volunteer, the PNE-ARDS patient, and the sep-ARDS patients. **(E)** Heat map of WGBS (left), RNA-seq (middle), and scRNA-seq (right) demonstrating single-cell contribution of genes specifically expressed in the PNE-ARDS patient and sep-ARDS patients. ARDS, acute respiratory distress syndrome; PBMC, peripheral blood mononuclear cell; cDCs, classical dendritic cells; MDSCs, myeloid-derived suppressive cells; Macs, macrophages; MLPCs, multi-lineage progenitor cells; scRNA-seq, single-cell RNA sequencing.

In the current study, we sought to gain more insight into the immune responses of ARDS patients with different etiologies during the course of the disease. To this end, we performed scRNA-seq (10× Genomics) of peripheral blood single nuclei from one PNE-ARDS patient, two sep-ARDS patients, and one healthy volunteer (Figure 1A). Clustering analysis captured a total of 15,889 cells in 12 different cell clusters identified as defined cell types based on known markers (Figure 1B, Supplementary Table S2). Known cell marker genes were specifically expressed in all of these cell type clusters (Jiang et al., 2020a) (Figure 1C, Supplementary Table S3). Further comparison of the cell composition among control, sep-ARDS, and PNE-ARDS revealed that the dynamics of neutrophils, macrophages (Macs), classical dendritic cells (cDCs), myeloid-derived suppressive cells (MDSCs), and CD8^+^ T cells varied significantly among groups of different samples, with neutrophils and cDCs being more abundant and Macs significantly less abundant in sep-ARDS patients; MDSCs were highly enriched only in sep-ARDS, and CD8^+^ T cells were more abundant in PNE-ARDS (Figure 1D). Differential expression analysis of RNA-seq, WGBS, and scRNA-seq data revealed the contribution of genes analyzed at the single-cell level that were expressed in the healthy volunteer, sep-ARDS patients, and PNE-ARDS patient (Figure 1E). These data suggest that the obtained changes in bulk-seq levels and scRNA-seq of genes may mediate the onset and progression of ARDS. Therefore, we initially mapped PBMCs from ARDS patients and explored alterations in peripheral circulation associated with different ARDS types relative to normal conditions. Ultimately, changes in gene levels by bulk-seq and scRNA-seq revealed potential factors mediating the development of ARDS.

### 3.2 ARDS-associated CD8^+^ T cell subpopulations

**FIGURE 2:**
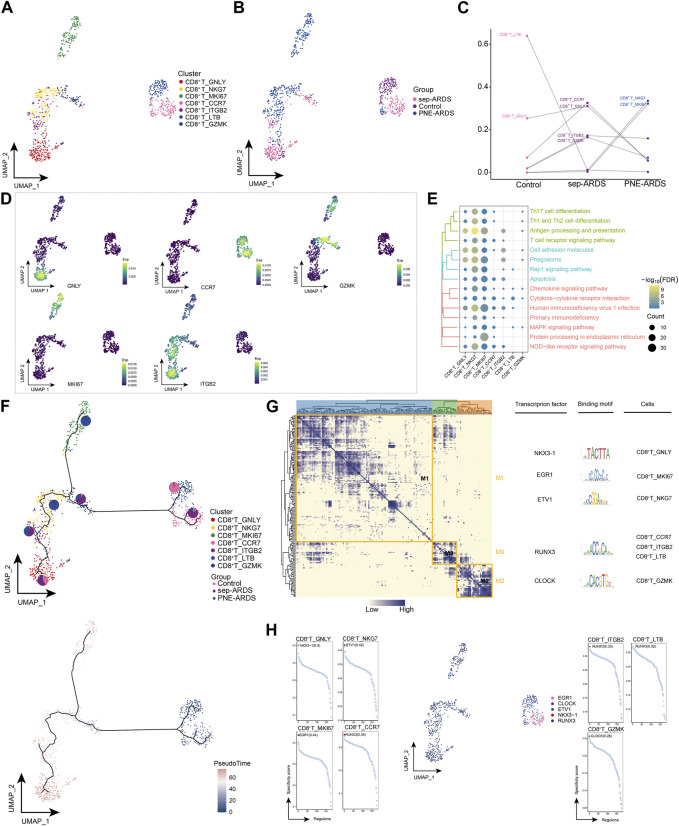
Acute respiratory distress syndrome (ARDS)-associated CD8^+^ T cell subpopulations. **(A)** Single-cell atlas illustrating CD8^+^ T cell subpopulations. **(B)** Single-cell atlas showing CD8^+^ T cell subpopulations in control, septic (sep)-ARDS, and pneumonic (PNE)-ARDS. **(C)** Dotted line graph indicating differences in CD8^+^ T cell subset abundance among control, sep-ARDS, and PNE-ARDS. **(D)** Series of single-cell atlases illustrating markers for specific cell subpopulations. **(E)** Heat map highlighting signaling pathways significantly activated by CD8^+^ T cell subpopulations. **(F)** Pseudo-time analysis illustrating proposed chronological trajectory and proposed chronological values of CD8^+^ T cells from healthy to diseased, with pie charts representing the proportion of each subpopulation in control, sep-ARDS, and PNE-ARDS. **(G)** Transcript expression in CD8^+^ T cells in patients with ARDS with different etiologies (co-expression modules of factors). Left: identification of regulator modules according to the CSI matrix of regulators. Middle: representative transcription factors and their binding patterns in the modules. Right: cell subpopulations where transcription factors are located. **(H)** Series of scatter plots showing the transcription factors regulating the CD8^+^ T cell subpopulations. ARDS, acute respiratory distress syndrome; UMAP, uniform manifold approximation and projection.

In the PNE-ARDS patient, CD8^+^ T cells were more abundant and their cytotoxic effect hypothetically cleared viral infections; hence, we re-clustered CD8^+^ T cells and obtained seven different cell subpopulations (Figure 2A). As shown in Figure 2B, the CD8^+^ T subpopulations from different ARDS types were heterogeneous. Further comparison of the subpopulations observed in the healthy volunteer, sep-ARDS patients, and the PNE-ARDS patient showed that CD8^+^ T_LTB cells were less abundant in sep-ARDS patients and the PNE-ARDS patient and CD8^+^ T_ITGB2, CD8^+^ T_CCR7, and CD8^+^ T_GZMK were more abundant in sep-ARDS patients, while CD8^+^ T_NKG7 and CD8^+^ T_MKI67 were more abundant in the PNE-ARDS patient, relative to the control (Figure 2C). This further highlights the inter-patient heterogeneity of CD8^+^ T subpopulations. The marker gene expression for the cell clusters presenting significant changes between the three conditions was mapped on a single-cell atlas (Figure 2D). Enrichment analysis revealed that the CD8^+^ T cell subclusters significantly activated apoptosis, antigen processing and presentation, T cell receptor signaling pathways, associated inflammatory pathways, and immune-related pathways (Figure 2E, Supplementary Table S4). We also explored the developmental trajectory of CD8^+^ T cells and clarified the polarization from healthy to sep-ARDS patients and the PNE-ARDS patient during the course of ARDS using pseudo-time analysis (Figure 2F). In addition to this, the GRN, with transcription factors (TFs) as the fulcrum, was organized into three modules (Figure 2G), directing cell fate selection by regulating specific gene expression of ARDS-specific CD8^+^ T cells through TFs such as NKX3-1, EGR1, ETV1, RUNX3, and CLOCK (Figure 2H). These results suggest that multiple CD8^+^ T cell subpopulations exist and are heterogeneous in ARDS patients with different etiologies. Among them, some of these specific subpopulations are clearly involved in apoptosis, inflammatory pathways, and immune-related pathways, which in turn induce ARDS.

### 3.3 ARDS-associated neutrophil subpopulations

**FIGURE 3:**
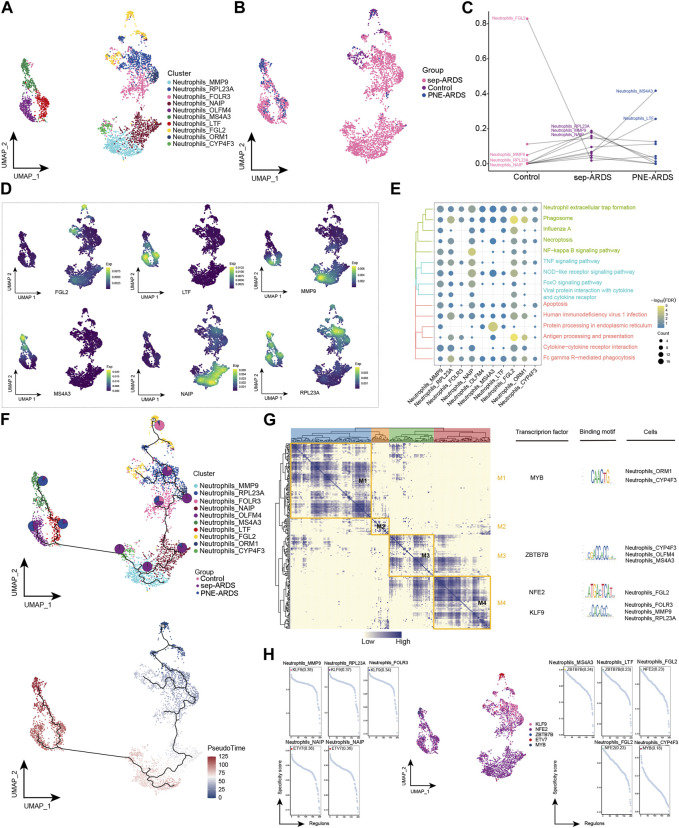
Acute respiratory distress syndrome (ARDS)-associated neutrophil subpopulations. **(A)** Single-cell atlas showing neutrophil subpopulations. **(B)** Single-cell atlas showing the neutrophil subpopulations in control, septic (sep)-ARDS, and pneumonic (PNE)-ARDS. **(C)** Dotted line graph indicating differences in neutrophil subpopulation abundance among control, sep-ARDS, and PNE-ARDS. **(D)** Series of single-cell atlases illustrating markers for specific cell subpopulations. **(E)** Heat map highlighting signaling pathways significantly activated by neutrophil subpopulations. **(F)** Pseudo-time analysis showing proposed chronological trajectory and proposed chronological values of neutrophils from healthy to diseased, with pie charts representing the proportion of each subpopulation in control, sep-ARDS, and PNE-ARDS. **(G)** Co-expression of transcription factors in neutrophils from patients with ARDS with different etiologies. Left: identification of regulator modules according to the CSI matrix of regulators. Middle: representative transcription factors and their binding patterns in the modules. Right: cell subpopulations where transcription factors are located. **(H)** Series of scatter plots showing transcription factors regulating the neutrophil subpopulations. ARDS, acute respiratory distress syndrome; UMAP, uniform manifold approximation and projection.

A key feature of ARDS is the accumulation of neutrophils in the pulmonary microvasculature, interstitium, and alveolar spaces (Williams and Chambers, 2014). We observed significantly elevated neutrophil levels in sep-ARDS patients. Therefore, neutrophils were re-clustered and 10 different cell subpopulations were obtained (Figure 3A). Consistent with a previous study (Williams and Chambers, 2014), neutrophils were abundant in patients with sep-ARDS (Figure 3B). Notably, Neutrophils_FGL2 was significantly less abundant in ARDS patients, Neutrophils_RPL23A, Neutrophils_MMP9, and Neutrophils_NAIP were more abundant in sep-ARDS patients, while Neutrophils_MS4A3 and Neutrophils_LTF were more abundant in the PNE-ARDS patient (Figure 3C), thus tentatively demonstrating the heterogeneity of neutrophil subpopulations among patients. Moreover, markers for these specific subpopulations mapped to single cell profiles (Figure 3D). By further understanding the role played by different subpopulations of neutrophils in ARDS progression, we found that different subpopulations clearly activated apoptosis, related inflammatory pathways, and immune-related pathways (Figure 3E). In addition, exploration of neutrophil BP revealed that neutrophil subpopulations significantly activated some oxidative stress responses (Supplementary Figure S1, Supplementary Table S4). Subsequently, the evolutionary trajectory of neutrophil subpopulations was explored, and we observed that the developmental trajectory of neutrophils evolved from Neutrophils_FGL2 (starting point), toward Neutrophils_RPL23A to Neutrophils_MS4A3 (Figure 3F). In addition, the GRN of the neutrophil subpopulation was constructed, and the regulators were hierarchically clustered according to the connection specificity index (CSI) to rank the importance of regulators and mitigate the effect of non-specific interactions. We observed that the TFs pivoted on MYB, ZBTB7B, NFE2, and K2F9 GRN were organized into four modules (Figure 3G), which in turn regulated specific gene expression in ARDS-specific neutrophils (Figure 3H). In conclusion, our results suggest the presence of multiple neutrophil subpopulations in ARDS patients, some of which are clearly involved in apoptosis, inflammatory pathways, and immune-related pathways; more importantly, they suggest the significant involvement of neutrophils in oxidative stress responses that may contribute to ARDS development, suggesting a possible reciprocal promotion of cell death and inflammation caused by oxidative stress in neutrophils.

### 3.4 ARDS-associated cDC subpopulations

**FIGURE 4:**
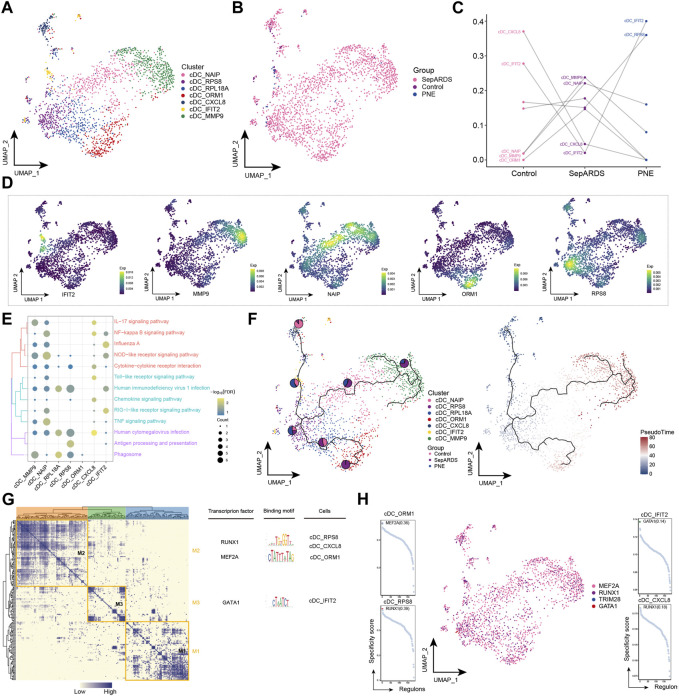
Acute respiratory distress syndrome (ARDS)-associated classical dendritic cell (cDC) subpopulations. **(A)** Single-cell atlas illustrating cDC subpopulations. **(B)** Single-cell atlas showing cDC subpopulations in control, septic (sep)-ARDS, and pneumonic (PNE)-ARDS. **(C)** Dotted line graph indicating differences in cDC subpopulation abundance among control, sep-ARDS, and PNE-ARDS. **(D)** Series of single-cell atlases illustrating markers for specific cell subpopulations. **(E)** Heat map indicating signaling pathways significantly activated by cDC subpopulations. **(F)** Pseudo-time analysis highlighting proposed chronological trajectory and proposed chronological values of cDCs from healthy to diseased, with pie charts representing the proportion of each subpopulation in control, sep-ARDS, and PNE-ARDS. **(G)** Co-expression of transcription factors in cDCs from patients with ARDS with different etiologies. Left: identification of regulator modules according to the CSI matrix of regulators. Middle: representative transcription factors and their binding patterns in the modules. Right: cell subpopulations in which transcription factors are located. **(H)** Series of scatter plots showing transcription factors regulating the cDC subpopulations. ARDS, acute respiratory distress syndrome; UMAP, uniform manifold approximation and projection.

It is generally agreed that cDCs play a key role in initiating acquired immunity and host resistance to infection (Hirako et al., 2019); here, cDC re-clustering yielded seven cell subpopulations (Figure 4A), almost all of which were abundant in the sep-ARDS patient group (Figure 4B). Specific cDC subpopulations in ARDS patients with different etiologies were identified by analyzing the changes in abundance of different subpopulations between the control, sep-ARDS patients, and the PNE-ARDS patient (Figure 4C). We observed that cDC_MMP9, cDC_NAIP, and cDC_ORM1 were increased in abundance in sep-ARDS patients compared with those in the healthy volunteers. Furthermore, the marker expression for these specific cDC subpopulations was mapped in the single-cell atlas (Figure 4D). These specific cDC subpopulations were significantly involved in inflammatory pathways as well as immune-related pathways (Figure 4E, Supplementary Table S4). The evolutionary trajectory of the cDC cell subpopulation started from healthy controls and differentiated towards sep-ARDS patients and PNE-ARDS patients (Figure 4F). The GRN branched by TFs such as RUNX1, MEF2A, and GATA1 was organized into three modules (Figure 4G), and these TFs have been shown to regulate specific gene expression in ARDS-specific cDCs (Figure 4H). These results suggest an increased abundance of specific CDC subpopulations in sep-ARDS patients compared to that in healthy volunteers and that these specific CDC subpopulations are clearly involved in inflammatory pathways and immune-related pathways that may mediate the development of ARDS.

### 3.5 ARDS-associated mac subpopulation

**FIGURE 5:**
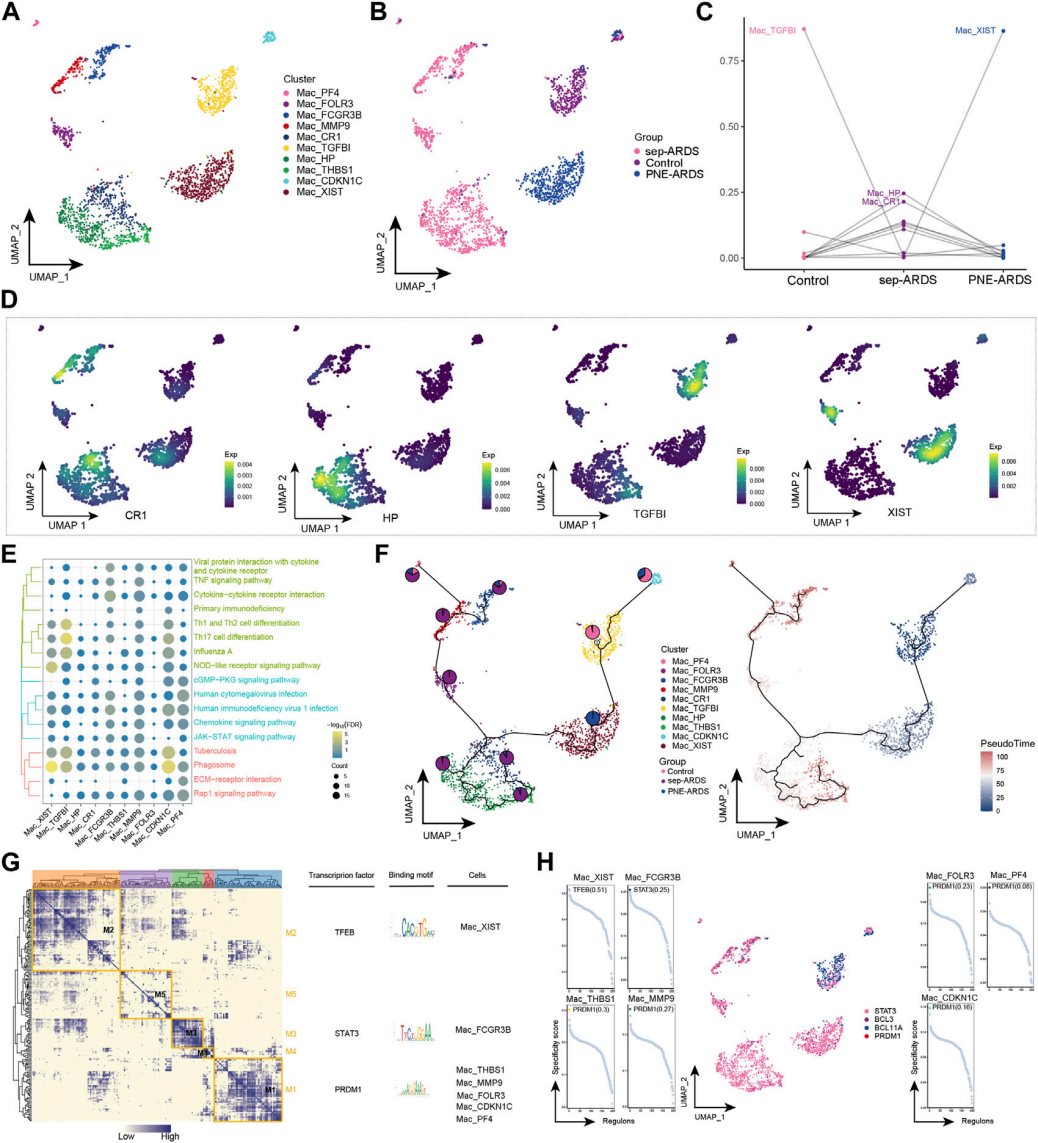
Acute respiratory distress syndrome (ARDS)-associated macrophage (Mac) subpopulations. **(A)** Single-cell atlas showing Mac subpopulations. **(B)** Single-cell atlas showing Mac subpopulations in control, septic (sep)-ARDS, and pneumonic (PNE)-ARDS. **(C)** Dotted line graph indicating differences in Mac subpopulation abundance among control, sep-ARDS, and PNE-ARDS. **(D)** Series of single-cell atlases showing markers for specific cell subpopulations. **(E)** Heat map indicating signaling pathways significantly activated by Mac subpopulations. **(F)** Pseudo-time analysis illustrating proposed chronological trajectory and proposed chronological values of Macs from healthy to diseased, with pie charts representing the proportion of each subpopulation in control, sep-ARDS, and PNE-ARDS. **(G)** Co-expression of transcription factors in Macs from patients with ARDS with different etiologies. Left: identification of regulator modules according to the CSI matrix of regulators. Middle: representative transcription factors and their binding patterns in the modules. Right: cell subpopulations in which transcription factors are located. **(H)** Series of scatter plots showing transcription factors regulating the Mac subpopulations. ARDS, acute respiratory distress syndrome; UMAP, uniform manifold approximation and projection.

The role of Macs in the development of inflammatory responses is subtle. In general, they have a pro-inflammatory role in the early stages and an anti-inflammatory role in the late stages of the inflammatory response (Huang et al., 2018). Notably, the abundance of Macs was significantly reduced in sep-ARDS and increased in PEN-ARDS. Subsequent Mac re-clustering yielded ten cell subpopulations (Figure 5A), which were mapped in different sample groups (Figure 5B). By analyzing the change in abundance of the different subpopulations between control, sep-ARDS patients, and the PNE-ARDS patient, specific subpopulations were associated with ARDS (Figure 5C). Mac_TGFBI was the most abundant in the healthy control and significantly lower in sep-ARDS patients; Mac_XIST was significantly more abundant in the PNE-ARDS patient than in the healthy control. The marker expression for specific Mac subpopulations was mapped in the single-cell atlas (Figure 5D). In addition, enrichment analysis revealed that these specific Mac subpopulations were significantly involved in the JAK/STAT signaling pathway, IL-17 signaling pathway, inflammatory pathway, and immune-related pathways (Figure 5E, Supplementary Table S4). The pseudo-time analysis further explored the evolutionary trajectory of Mac subpopulations and the polarization process from healthy individuals to sep-ARDS patients and PNE-ARDS patients during the course of ARDS was clarified (Figure 5F). The GRN branched by TFs such as TFEB, STAT3, and PRDM1 was organized into five modules (Figure 5G), and these TFs have been shown to regulate specific gene expression in Mac (Figure 5H).

### 3.6 ARDS-associated MDSC subpopulation

**FIGURE 6:**
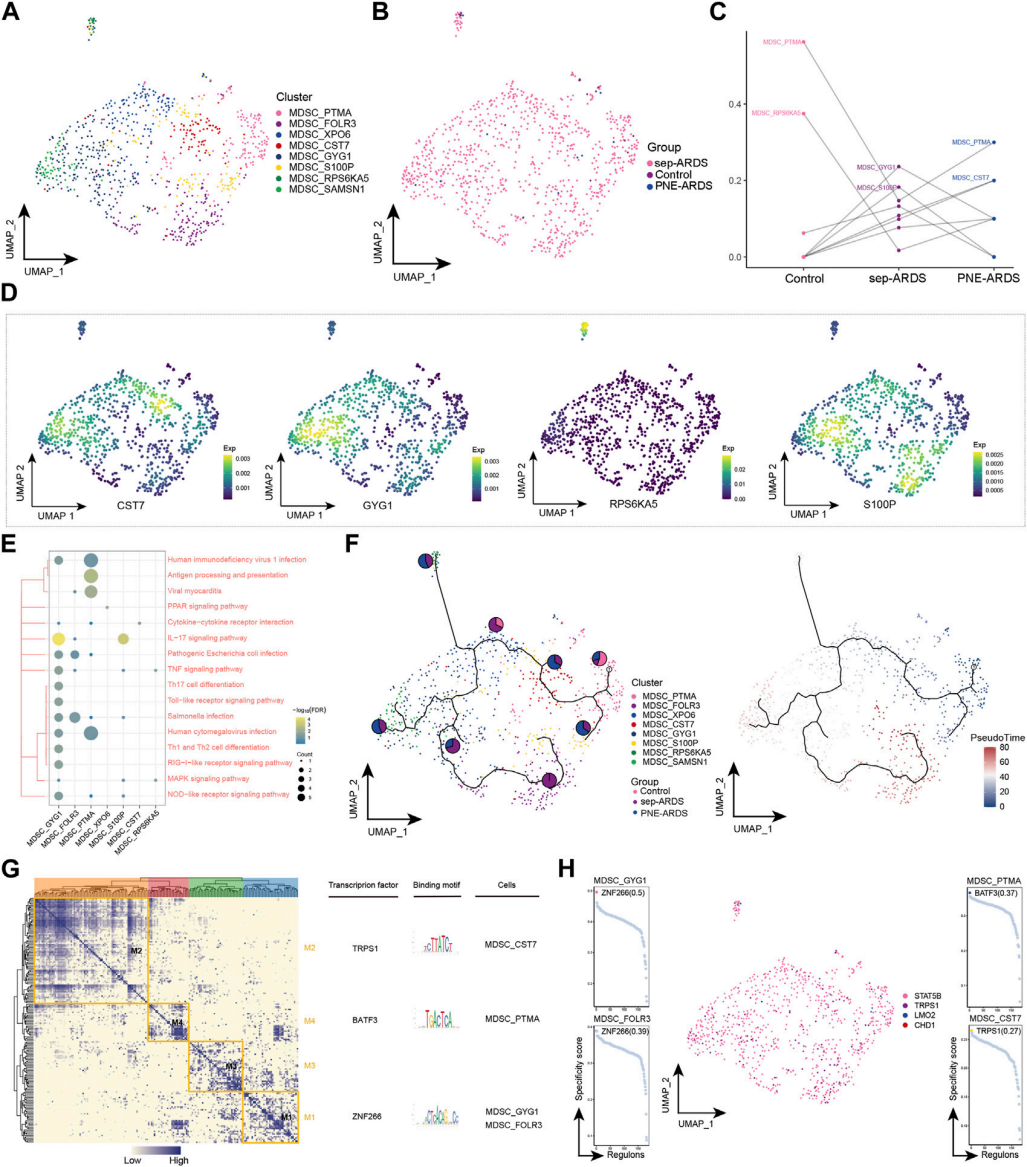
Acute respiratory distress syndrome (ARDS)-associated myeloid-derived suppressor cell (MDSC) subpopulations. **(A)** Single-cell profiles of MDSC subpopulations. **(B)** Single-cell profiles of MDSC subpopulations in control, septic (sep)-ARDS, and pneumonic (PNE)-ARDS. **(C)** Dotted line plots indicate differences in MDSC subpopulation abundance among control, sep-ARDS, and PNE-ARDS. **(D)** Series of single-cell profiles highlighting markers for specific cell subpopulations. **(E)** Heat map indicating signaling pathways significantly activated by MDSC subpopulations. **(F)** Pseudo-time analysis illustrating proposed chronological trajectories and proposed chronological values of MDSCs from healthy to diseased, with pie charts representing the proportion of each subpopulation in control, sep-ARDS, and PNE-ARDS. **(G)** Transcriptional co-expression module of the factors. Left: identification of regulator modules according to the CSI matrix of regulators. Middle: representative transcription factors and their binding patterns in the modules. Right: cell subpopulations where transcription factors are located. **(H)** Series of scatter plots showing transcription factors regulating the MDSC subpopulations. ARDS, acute respiratory distress syndrome; UMAP, uniform manifold approximation and projection.

MDSCs are a heterogeneous population of myeloid cells characterized by enhanced immunosuppression in chronic infections and cancers (Kumar et al., 2016). In the present study, MDSCs were observed to be highly enriched only in sep-ARDS patients and warrant further exploration. Eight cell subpopulations were obtained by re-clustering MDSCs (Figure 6A), and almost all of these subpopulations were abundant in the sep-ARDS group (Figure 6B). Specific ARDS etiologies could be identified by analysis of the changes in MDSC abundance differences between control, sep-ARDS patients, and the PNE-ARDS patient (Figure 6C). And the marker expression of specific MDSC subpopulations was mapped in a single-cell atlas (Figure 6D). Enrichment analysis revealed that these specific MDSC subpopulations were significantly involved in IL-17 signaling pathways, inflammatory pathways, and immune-related pathways (Figure 6E, Supplementary Table S4). The evolutionary trajectory of MDSC cell subpopulations was explored and differentiation was observed starting from healthy controls, towards sep-ARDS patients and PNE-ARDS patients (Figure 6F). The GRN branched by TFs such as TRPS1, BATF3, and ZNF266 was organized into four modules (Figure 6G), and these TFs were shown to regulate specific gene expression in ARDS-specific MDSC (Figure 6H).

## 4 Discussion

In this study, differences in microenvironmental cells in whole blood collected from sep- and PEN-ARDS patients as well as healthy controls were explored. We combined multi-omics sequencing data on PBMCs with scRNA-seq, RNA-seq, and WGBS to identify alterations in peripheral circulation associated with different types of ARDS as well as changes in gene levels by bulk-seq and scRNA-seq to reveal potential factors mediating ARDS development. Several specific cell subpopulations identified in the study were previously known to be associated with ARDS. For example, infiltration of neutrophils, macrophages, and lymphocytes (Hu et al., 2020). The current study suggests that the global landscape of patients with PNE-ARDS and sep-ARDS can be explored at the cellular and molecular level using the finer resolution provided by scRNA-seq technology.

T cells are immune effector cells that fight viral infections, and patients with sepsis experience chronic immunosuppression resulting in increased susceptibility to infections normally controlled by T cells (Danahy et al., 2016). In addition, previous studies have shown that sepsis is associated with a reduction in CD8^+^ T cell numbers and their functional responses (Guo et al., 2021). Our study showed that CD8^+^ T cell numbers were significantly reduced in sep-ARDS, suggesting a mechanism driving immunosuppression. Enrichment analysis revealed that CD8^+^ T cells were significantly involved in the apoptotic signaling pathway, thus suggesting that CD8^+^ T cells undergo apoptosis, leading to a significant decrease in numbers. However, to the best of our knowledge, few studies have determined the effect of ARDS on CD8^+^ T cells. In conclusion, these results suggest that T cell-mediated immune damage in ARDS may be a key factor contributing to the process in ARDS, a concept that warrants further investigation.

Neutrophils are innate immune cells that have a short life span after leaving the bone marrow and exist in a resting, sensitized, or active state. These leukocytes are the major players in innate immunity as they are the first innate leukocytes recruited during infection (Lamichhane and Samarasinghe, 2019). The primary function of neutrophils is to remove pathogens and debris through phagocytosis (Rosales, 2020). They also have a unique set of other immune roles, such as releasing NETs to inactivate viral infection (Barr et al., 2018) and producing cytokines to limit viral replication (Lamichhane and Samarasinghe, 2019). We observed a significant increase in neutrophil numbers in sep-ARDS, which is consistent with previous reports. Nicolai et al. showed that ARDS patients exhibited neutrophil aggregation in the blood and that this changed with the severity of the disease (Nicolai et al., 2020). Notably, neutrophil subpopulations were also found to be involved in inflammation-related pathways and cytokine-related pathways, thus suggesting that other immune cells produce pro-inflammatory cytokines and chemokines in sep-ARDS. This, in turn, leads to excessive neutrophil infiltration into the tissues, which allows activated neutrophils to further aggravate tissue inflammation and injury in sepsis. In addition, innate immune cells are a major source of reactive oxygen species (ROS) and the inflammatory response in the lungs enhances the permeability of neutrophils to penetrate the endothelial-epithelial barrier and release pro-inflammatory cytokines (Li et al., 2020).

The role of cDCs in human diseases such as autoimmune diseases has been thoroughly studied, but data on the role of dendritic cells in ARDS are limited. During the development of ARDS, cDCs can play a direct role in abnormal immune responses (Elsayh et al., 2013; Weber et al., 2015). In our study, cDCs were found to be significantly enriched in sep-ARDS patients, and cDCs might secrete cytokines to manipulate lymphocyte function; therefore, modulating and improving cDC function might improve immune function in sep-ARDS. In addition, septic cDCs display abnormal cytokine secretion, leading to a state of immune tolerance. Several molecules have been shown to represent promising targets for improving cDC function and extending their lifespan during sepsis progression (Kumar, 2018; Qian and Cao, 2018). Our studies have shown changes in the number of dendritic cells, their subpopulation differentiation, and their associated functions in sep- and PNE-ARDS. Therefore, further investigation and elucidation of the new mechanisms underlying cDC alterations in ARDS could contribute to the development of new antimicrobial drugs.

Macs are important effector cells, involved in all phases of ARDS (Chen et al., 2020). In the inflammatory state, circulating monocytes are recruited into tissues and polarized into different phenotypes (M0, M1, M2), and macrophages in tissues (e.g., alveolar macrophages) are similarly polarized into different phenotypes, thus participating in the inflammatory process or tissue remodeling (Arora et al., 2018; Atri et al., 2018). Macs polarize into different functional phenotypes as a response to specific microenvironmental stimuli and signals. Among them, M2 is the phenotype that exerts suppressive inflammatory overreaction and negative immunomodulatory effects, which can be polarized into M2a, M2b, M2c, and M2d subtypes when stimulated by IL-4/IL-13, LPS/immunocomplexes, IL-13/TGF-β, and tumor-associated factors, respectively (Lu et al., 2013; Murray, 2017; Arora et al., 2018; Orecchioni et al., 2019). Numerous signaling pathways are involved in macrophage polarization during the course of ARDS, and the JAK/STAT pathway is one of the most important ones (Platanitis and Decker, 2018; Jiang et al., 2020b). Notably, our study showed that Mac subpopulations were significantly involved in the JAK/STAT signaling pathway. In addition, targeting Mac polarization as an ARDS therapeutic has been shown to be effective and promising in several preclinical studies (Bittencourt-Mernak et al., 2017; Tu et al., 2017; Wang et al., 2019). Limiting excessive pro-inflammatory responses and tissue damage by regulating Mac activation and polarization and balancing excessive fibrosis as well as tissue repair represent novel potential therapeutic targets for ARDS.

MDSCs are excreted from the bone marrow as functionally immature cells. They mature into mononuclear MDSCs (mMDSCs) and granulocyte MDSCs (gMDSCs) in response to signals from the microenvironment. They inhibit the T cell cycle and immune checkpoints, downregulate T cell receptors, and recruit Tregs. MDSCs also inhibit the activity of other immune cells by producing ROS and RNS, degrading arginine, and producing anti-inflammatory factors such as TGF-β and IL-10 (Schrijver et al., 2019). Recent studies have shown that granulocyte colony-stimulating factor (G-CSF) and granulocyte-macrophage colony-stimulating factor (GM-CSF), a major driver of MDSCs recruitment and differentiation, is abundant in the lungs of COVID-19 patients. A recent analysis of MDSCs in 128 SARS-CoV-2-infected patients showed a very high frequency of MDSCs, especially in critically ill patients with concomitant ARDS, and it is very likely that the immunosuppressive function of MDSCs impedes viral clearance and increases disease severity (Sacchi et al., 2020). In the present study, MDSCs were shown to be significantly enriched in patients with sep-ARDS and MDSC subpopulations were significantly involved in the IL-17 signaling pathway and antigen-presentation signaling. In addition, in GRN, basic leucine zipper ATF-like transcription factor 3 (BATF3) has been shown to exhibit a regulatory role in MDSC subpopulations, and although the regulatory role of BATF3 in influenza A has been demonstrated (Lee et al., 2021), its role in ARDS has not been clarified. Therefore, our study suggests that MDSCs may be a valuable target for therapeutic intervention in ARDS patients (Kolahian et al., 2016).

However, a limitation of this study is that although immune cells in sep-ARDS and PNE-ARDS have an enhanced inflammatory profile compared to those in healthy controls, the mechanisms underlying the different immune responses in patients with sep-ARDS and PNE-ARDS have not been clarified. In addition, because PBMC samples were collected in this study and the sample size was small, future studies with larger sample sizes using lung tissue or alveolar lavage fluid are needed to further characterize single-cell transcription in different ARDS populations.

In conclusion, our study suggests that CD8^+^ T cells, neutrophils, cDC, Macs, and MDSCs of ARDS patients are key PBMC populations involved in the disease process and that these key cell populations undergo cellular and intermolecular changes that may contribute to disease progression.

## Data Availability

The datasets presented in this study can be found in online repositories. The names of the repository/repositories and accession number(s) can be found below: National Genomics Data Center under HRA002203 and HRA002228.
