# Supplementary material for: Single-cell analysis reveals dysregulated inflammatory response in peripheral blood immunity in patients with acute respiratory distress syndrome
Source: Front Cell Dev Biol. 2023 May 22;11:1199122. doi: 10.3389/fcell.2023.1199122 (PMC10239863; doi:10.3389/fcell.2023.1199122)
Supplement: Supplementary file 5 [file Image1.pdf]

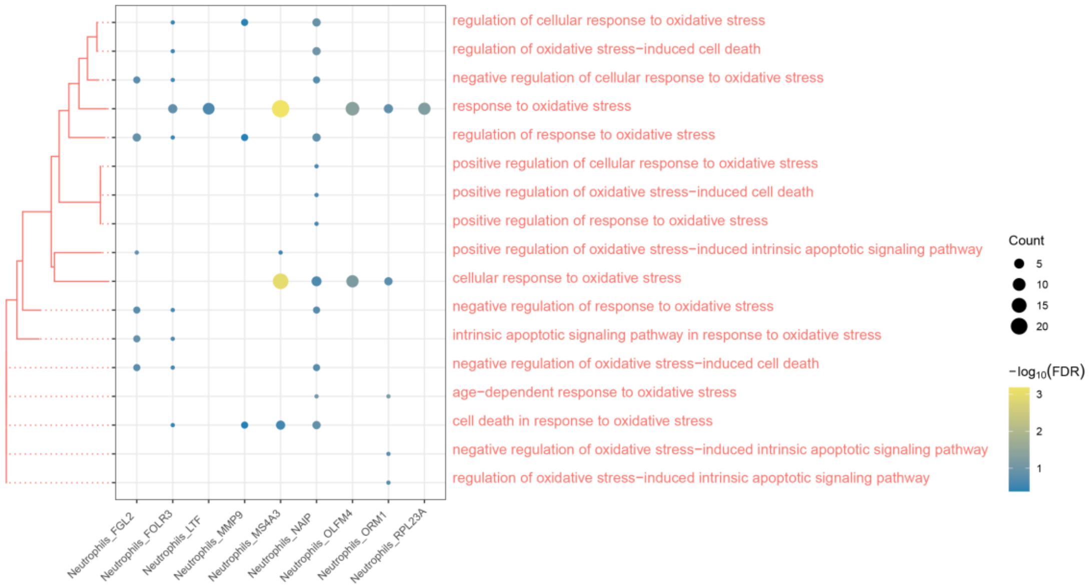

Supplementary Figure 1. Biological processes with significant involvement of neutrophil subpopulations.
